# Identification of chemosensitizing agents of colorectal cancer in *Rauvolfia vomitoria* using an NMR-based chemometric approach

**DOI:** 10.3389/fchem.2022.1069591

**Published:** 2023-01-06

**Authors:** Wei-Liang Cui, Dong-Xiao Guo, Ning Wang, Zhi-Fan Wang, Jian-Bo Ji, Xiao Wang, Chun-Guo Yang, Yong-Qiang Lin, Shu-Qi Wang

**Affiliations:** ^1^ Shandong Institute for Food and Drug Control, Jinan, Shandong, China; ^2^ Key Laboratory of Chemical Biology of Nature Products (Ministry of Education), School of Pharmaceutical Sciences, Cheeloo College of Medicine, Shandong University, Jinan, China; ^3^ Shandong Yifang Pharmaceutical Co., Ltd., Jinan, China; ^4^ NMPA Key Laboratory for Quality Evaluation of Gelatin Products, Jinan, China; ^5^ Shandong Engineering Research Center for Generic Technologies of Traditional Chinese Medicine Formula Granules, Jinan, China

**Keywords:** biochemometric, colorectal cancer, chemosensitization, Rauvolfia vomitoria, 1H NMR

## Abstract

Searching for new adjuvants of conventional chemotherapeutic approaches against colorectal cancer cells is extremely urgent. In current research, a non-targeted analytical approach was established by combining proton nuclear magnetic resonance spectroscopy with a chemometrics data mining tool to identify chemosensitizing agents from *Rauvolfia vomitoria*. This approach enabled the identification of potential active constituents in the initial fractionation process and provided their structural information. This strategy was validated by its application to *Rauvolfia vomitoria* extract exhibiting chemosensitizing activity on 5-fluorouracil against colorectal cancer cells. After the workflow, the biochemometrics analysis showed that at least 15 signals (Variable influence on projection (VIP) > 1) could have contributions in the differentiation of various fractions. Through systematic literature and database searches, we found that the most active fraction (fraction 7) exhibited the highest presence of sabazin-type and armaniline-type alkaloids, which were potential chemosensitizers as previously reported. To validate the results of the strategy, the effect of 5-FU and compounds isolated from fraction seven incubation on HCT-8 and LoVo cell vialibilty were evaluated. These results evidenced that compound *β*-carboline (**3**), 1-methyl-*β*-carboline (**4**), and lochnerine (**6**) could enhance the cytotoxicity of 5-fluorouracil against to Colorectal cancer cells. Besides, 21 compounds including two new compounds were isolated from *Rauvolfia vomitoria*. The experimental results verify the reliability of the method, and this approach provides a new and efficient tool to overcome some of the bottlenecks in natural products drug discovery.

## 1 Introduction

Colorectal cancer (CRC) is one of the leading causes of death worldwide (Rawla et al., 2019). In China, more than 0.37 million new cases of CRC, resulting in more than 0.19 million deaths occurred in 2015. Currently, surgery or chemotherapy is chosen for the treatment of colon cancer, depending on the tumor location and stage at diagnosis, as well as individual characteristics of the patients (Bao et al., 2020; de Albuquerque Ribeiro Gondinho et al., 2020). 5-Fluorouracil (5-FU) is one of preferred anti-CRC drugs and causes cell death by disturbing the metabolism of nucleosides (Dong et al., 2022). However, its effectiveness is influenced by the drug toxicity and development of resistance of CRC cells, which occurred in most CRC patients during their treatment course, necessitating the development of novel personalized drugs and drug combinations (Hu et al., 2016; Abdalla et al., 2021).

Application of combination strategies improved the response rates of CRC cells to 5-FU and other drugs up to 50% (Zhang et al., 2008). In a phase II clinical study, the clinical response of 5-FU against CRC increased to 24–74% when it is combined with oxaliplatin and irinotecan (Comba et al., 2001). Besides, chemosensitization of CRC cells to 5-FU by many compounds of natural sources have been observed (Abaza et al., 2016; Rani et al., 2017; de Oliveira et al., 2018; Yang et al., 2020). Hence, cancer researchers have endeavored to find new chemosensitizers of conventional chemotherapy from natural products.

Monoterpene indole alkaloids, consisting of monoterpene and indole moieties, belongs to a critical group of N-heterocyclic specialized metabolites and possessed extensive medicinal values and fascinating structural diversity. They have been shown to exhibit a variety of biological activities, such as cytotoxic and anti-inflammatory activities (Alves et al., 2009). Accumulated evidences have demonstrated the significant roles of monoterpene indole alkaloids in maintaining human health, such as apoptosis inducers and modulators of multidrug resistance-associated protein one and P-glycoprotein (Paterna et al., 2015; Paterna et al., 2016; Paterna et al., 2017; Paterna et al., 2018). *Rauvolfia vomitoria* Afzel. (RVA) is a traditional herbal medicine widely distributed in Yunnan, Guangzhou, and Guangxi provinces, China. It has a long history to treat hypertension, pain, epilepsy, high fever, and gastrointestinal diseases. Previous phytochemical studies of RVA have revealed the presence of a series of monoterpene indole alkaloids (Zhan et al., 2020a; Zhan et al., 2020b), making it a potential source of chemosensitizers. Establishment of a robust analytical technique for rapidly identify the chemosensitizers from RVA is meaningful.

Biochemometrics is a statistical approach used to infer candidates responsible for the activity in natural products-based drug discovery. Simultaneous profiling of multiple components was accomplished by metabolomics approaches, typically through the combination of chromatographic analysis with spectroscopic or spectrometric approaches (IR, UV, MS, or NMR detection) (Kellogg et al., 2016). Statistical tools, such as orthogonal partial least-squares discriminant analysis (OPLS-DA), added the biological activity as a supervision variable to the S-plot, which is used to visualize the covariance and the correlation structure between the X-variables and the predictive score t. Variables situated in the upper-right and lower-left of the S-plot are regarded as potential markers combining both high reliability and model influence.

In order to screen for effective chemosensitizing drugs, we designed a NMR-based chemometric strategy to simultaneously apply to two types of CRC cells, expecting to screen for chemosensitizing drugs targeting different tumor cells. To evaluate the reliability of NMR-based chemometric strategy, the isolation of RVA components was carried out for obtaining the chemosensitizers predicted by the model. In this research, the alkaloid components in RVA were characterized to identify potential chemosensitizer candidates.

## 2 Results and discussion

### 2.1 RVA extract chemosensitizes CRC cells to 5-FU

**FIGURE 1:**
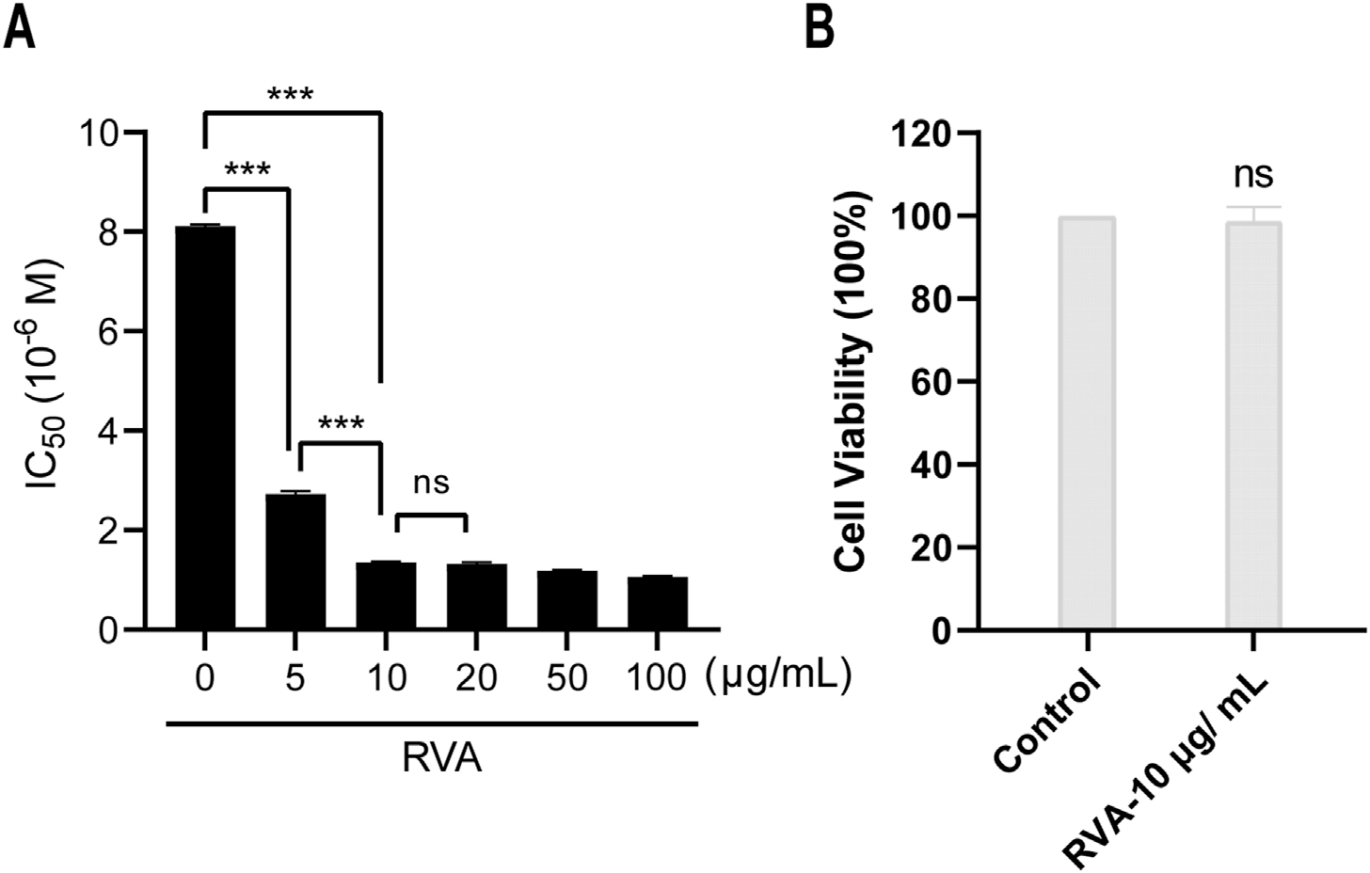
RVA sensitized HCT-8 cell line to 5-fluorouracil (5-FU) *via* a non-cytotoxic way. **(A)** HCT-8 cells were co-incubated with RVA and 5-FU at different concentrations for 72 h. The IC_50_ of 5-FU was evaluated by CCK–8 assay. **(B)** HCT-8 cell viability was measured after exposure to 10 μg/ml RVA for 72 h. The results shown are means ± SD; n = 3; *p < 0.05, **p < 0.01, ***p < 0.001.

**FIGURE 2:**
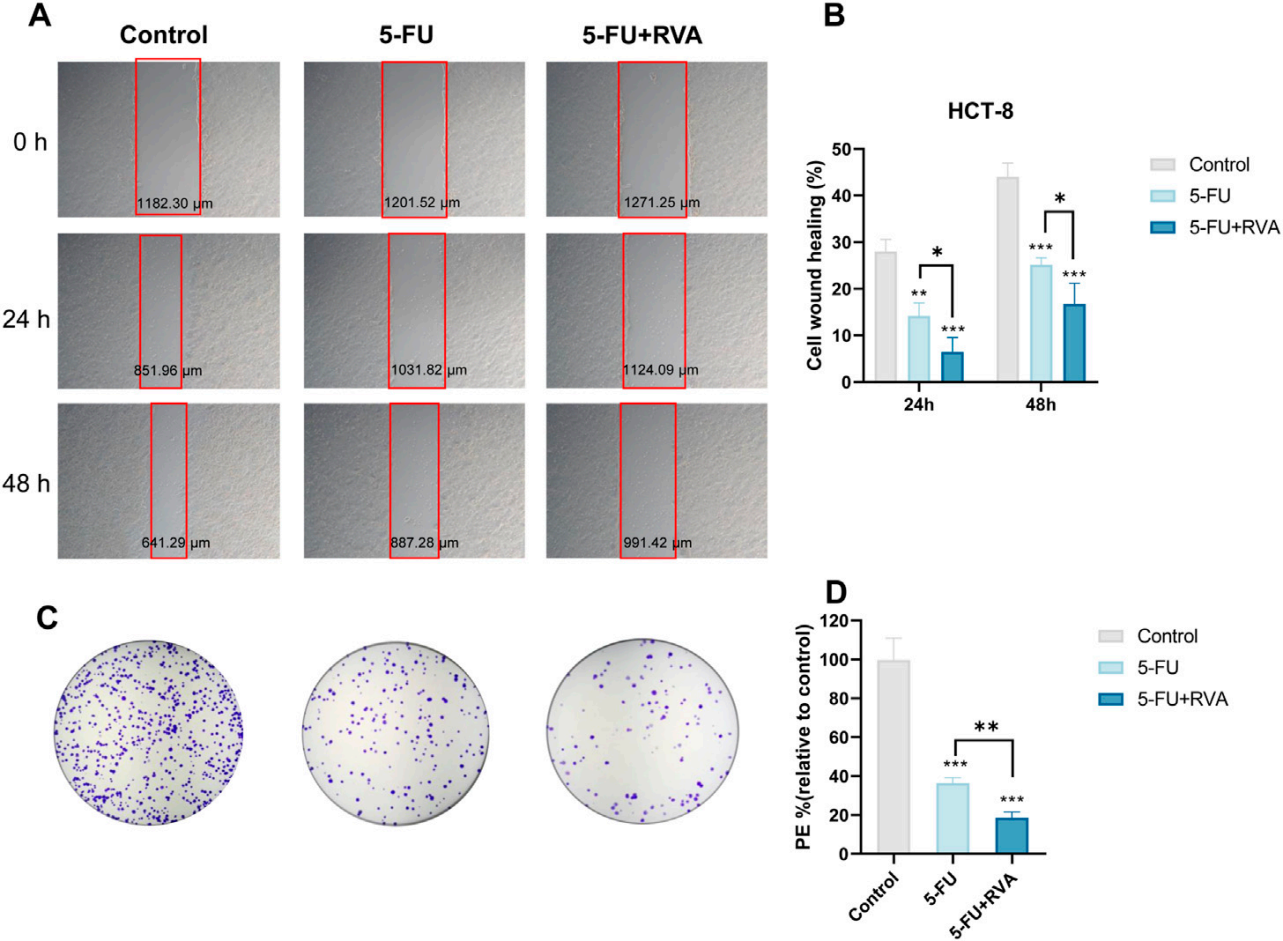
RVA enhances the anti–migration and anti-colony-forming activity of 5-FU. **(A)** HCT-8 cells treated with 10 μg/ml RVA and 1 μM 5-FU, co–incubated for 24 h and 48 h. Scratch healing photos of HCT-8 cells with different treatment at different time points under a brightfield microscope. **(B)** The migration ability of HCT-8 cells after different treatments at different time points. **(C)** After treatment with 5-FU (1 μM) in the presence or absence of RVA (10 μg/ml), the colony formation of HCT-8 cells was photographed. **(D)** The colony-forming ability of HCT-8 cells after different treatments. The results shown are means ± SD; n = 3; *p < 0.05, **p < 0.01, ***p < 0.001.

**FIGURE 3:**
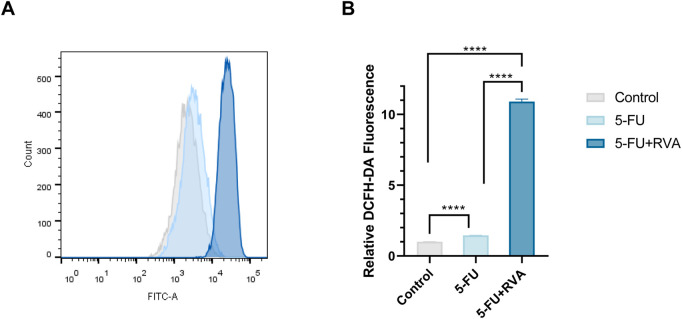
RVA enhances 5-FU-induced reactive oxygen species (ROS) accumulation. **(A)** HCT-8 cells treated with 5-FU (1 μM) or 5-FU combinated with RVA (10 μg/ml), co–incubated for 48 h. Flow cytometry was used to determine the fluorescence intensity after H2DCF–DA staining. **(B)** The results shown are means ± SD; n = 3; *p < 0.05, **p < 0.01, ***p < 0.001.

To investigate the chemosensitization effect of RVA, the IC_50_ value of 5-FU against HCT-8 cells were determined in the presence of different concentrations of RVA by 3-(4,5-dimethylthiazolyl-2)-2,5-diphenyltetrazolium bromide (MTT) assay. As shown in Figure 1A, RVA significantly lowers the IC_50_ values of 5-FU in a dose-dependent manner, while RVA alone (10 μg/ml) did not exhibit cytotoxicity against HCT-8 cells (Figure 1B). According to Figures 2A, B, RVA significantly enhanced the anti–migration activity of 5-FU. The antiproliferative activity of RVA against HCT-8 cells was then characterized using the colony formation (clonogenic) assay, which provides information about the colony-forming ability of cancer cells (Ruiz-Torres et al., 2021). For HCT-8 cells, combination treatment with 5-FU and RVA (10 μg/ml) caused a 49.1% reduction (Control: 567.3 ± 65.2; 5-FU only: 206.7 ± 16.5; 5-FU and RVA: 105.3 ± 17.6) in the number of colonies compared to cells treated with 5-FU alone (Figure 2C, D). Besides, we found that addition of RAV caused a significant increase in intracellular ROS levels in 5-FU treated HCT-8 cells, up to 7-fold compared to the 5-FU alone group (Figure 3). Collectively, the results confirmed that RVA could significantly enhance the anti-cancer activity of 5-FU, probably by, but not limited to, increasing the production of intracellular ROS.

### 2.2 Fractionation and biochemometric analysis of RVA extract

**FIGURE 4:**
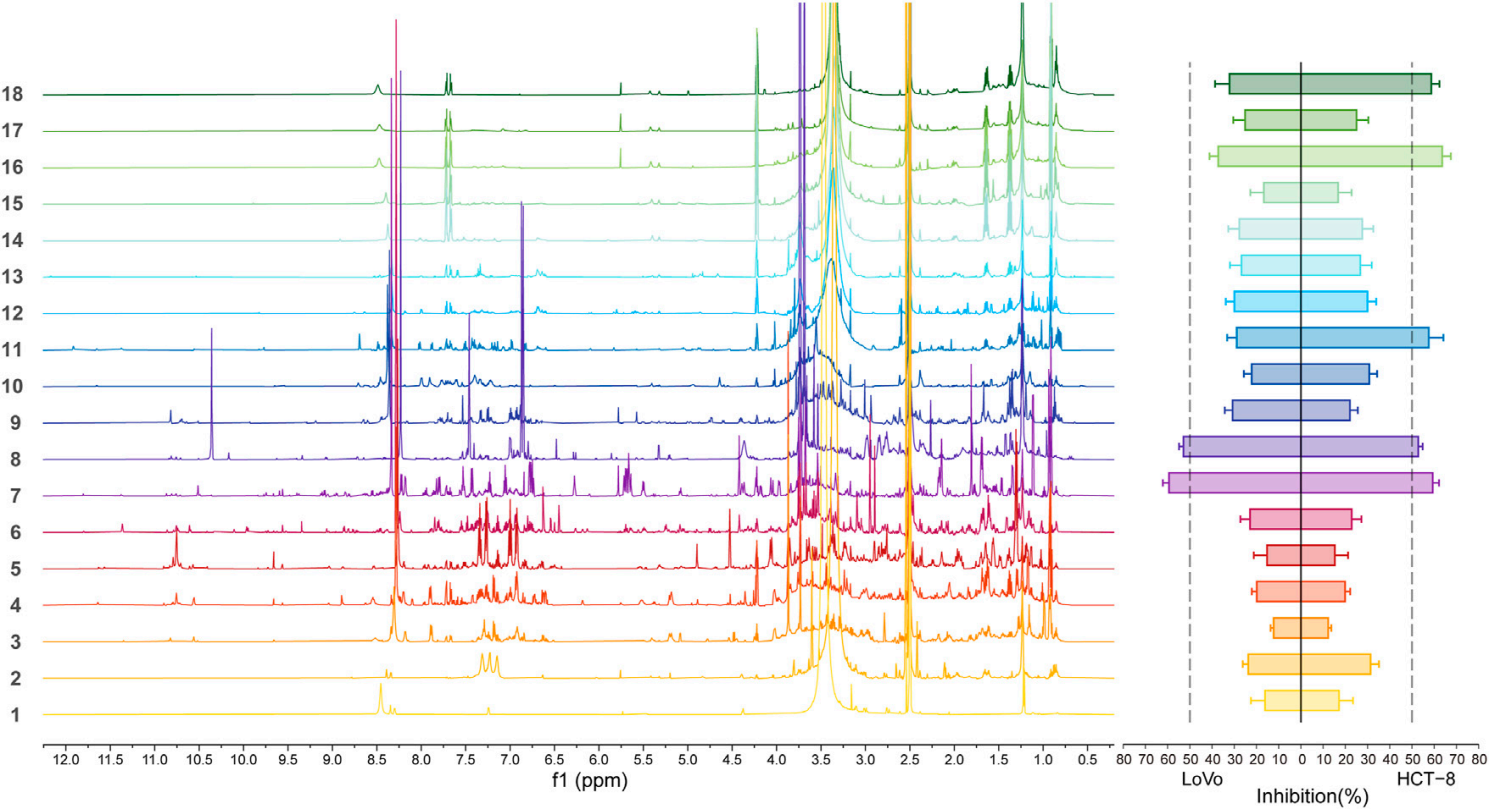
Representative ^1^H NMR (600 MHz; 6 mg/ml in DMSO-*d*_6_) spectra at 40°C (left) and two colorectal cells proliferation inhibitory activity profile (right) of the 18 fractions of RVA.

In order to fish the bioactive compounds out of RVA mixture, a biochemical chemometric strategy of ^1^H NMR was constructed. Firstly, RVA was fractionated in high performance liquid chromatography (HPLC) cartridges with a water/acetonitrile gradient, yielding 18 fractions. Given the rather large number of the samples, ^1^H NMR was selected for the analysis because of its shorter acquisition time compared to ^13^C or 2D experiments. ^1^H NMR spectra with good signal to noise ratios of the prepared fraction samples were then recorded. In this study, the spectrum was simplified to leave only the resonances of the bioactive constituents, by applying a correlation between spectroscopic data reflecting the concentration differences of contained compounds and concentration-dependent activity levels. Finally, the chemosensitization activity of 18 fractions were investigated at a concentration of 100 μg/ml and the efficacy of each fraction was expressed as a percentage of inhibition compared to the 5-FU only, which was set as 0% inhibition. Fractions seven was proved to possess the strongest activities against both cell lines. Plotting of the phased and baseline corrected ^1^H NMR spectra and the results of the inhibition tests provide first evidences of how certain resonances and activities fluctuate in parallel over consecutive fractions (Figure 4). The results demonstrated that ^1^H NMR chemical shifts characteristic for indole aromatic ring protons (*δ*_H_ 6.00–8.00) were observed in the active fraction three to fraction 18, indicating that the indole alkaloids are potential chemosensitizing agents.

**FIGURE 5:**
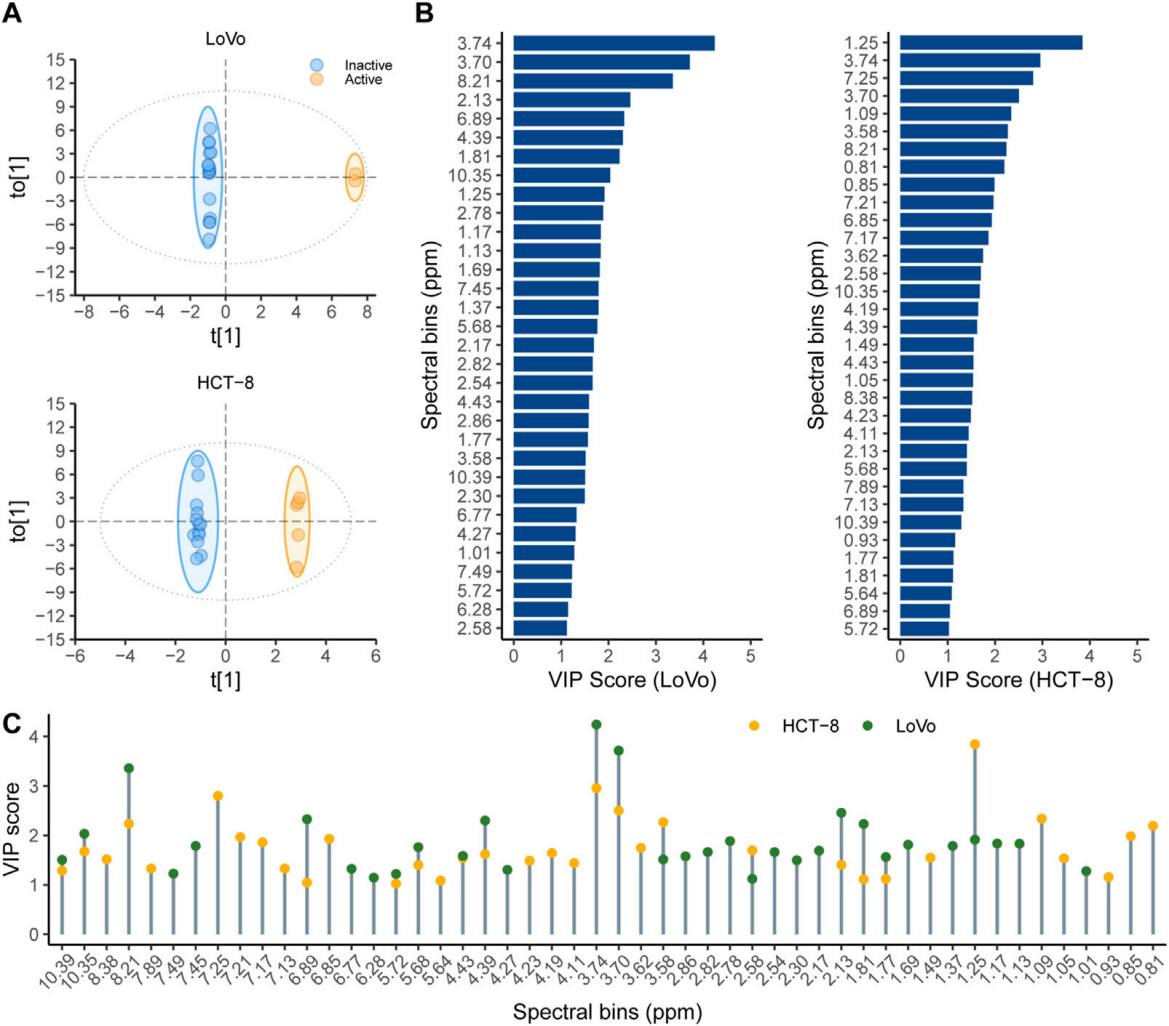
Multivariate analysis of the 18 fractions from RVA and their proliferation inhibitory activity against two colorectal cancer cells **(A)** OPLS-DA score plot showing different samples according to their activity. Samples that were able to inhibit the proliferation activity by 50% or more were labeled as active (orange), while those with inhibition below 50% were labeled as inactive (blue); **(B)** The highest VIP scores (VIP>1, ^1^H NMR bins) correspond to bins that contribute the most for the observed separation between the groups. **(C)** The comparation of VIP scores in different colorectal cancer cells, the orange circle represents the bins (ppm) with VIP score>1 in HCT-8 cells, the green circle represents the bins (ppm) with VIP score >1 in LoVo cells, and the two circles represent two cell strains shared bins (ppm).

Orthogonal partial least squares discriminant analysis (OPLS-DA) analysis was performed to get information of compounds with activities. The ^1^H NMR spectra were firstly divided into a series of 0.04 ppm bins and transformed to ASCII files using MestReNova (version 14, Mestrelab Reserach). The generated ASCII files were labeled in Microsoft EXCEL and then analyzed by SIMCA-P 14.0 (Umetrics, Umea, Sweden). In this research, samples were considered as active if they were able to increase the anti-cancer activity of 5-FU by 50%. The OPLS-DA, well suited for the classification of many types of biological data that have multi-collinear and noisy variables, was performed to correlate the ^1^H NMR spectra and chemosensitization effects of each fraction (Bylesjo et al., 2006; Casanova et al., 2020). Multivariate analysis of the NMR data of the 18 fractions from RVA and their proliferation inhibitory activity against two CRC cells were shown in Figure 5A. In LoVo cells, *R* ^2^ and Q^2^ values of statistical analysis model were 0.902 and 0.512, respectively. In HCT-8 cells, *R* ^2^ and Q^2^ values were 0.883 and 0.514, respectively. A value of Q^2^ larger than 0.5 is generally considered good. The OPLS-DA score plot showed a good discrimination between active and inactive fractions. The spectroscopic regions (bins) with the highest VIP scores (VIP >1) contribute most to the observed class separation, and are shown in the VIP score plot (Figure 5B). For the active fractions, the signals in 3.74, 3.70, and 8.21 ppm are readily distinguishable and regarded as important for their differentiation. Besides, the comparison of VIP scores in different CRC cells were shown in Figure 5C. The VIP scores of bins of 8.21, 6.89, 5.72, 3.74 and 3.70 ppm were high in analysis of both cells, indicating that the corresponding differential compounds were potential compounds with good activity in both cells.

### 2.3 Identification of active compounds in RVA extract

After identifying the signals (spectral bins) important for the activity according to the VIP scores, the subsequent step was to search for the bioactive compounds. Since the fraction seven exhibited the highest growth inhibition against both cell lines, it was chosen for analysis to identify the active compounds.

**FIGURE 6:**
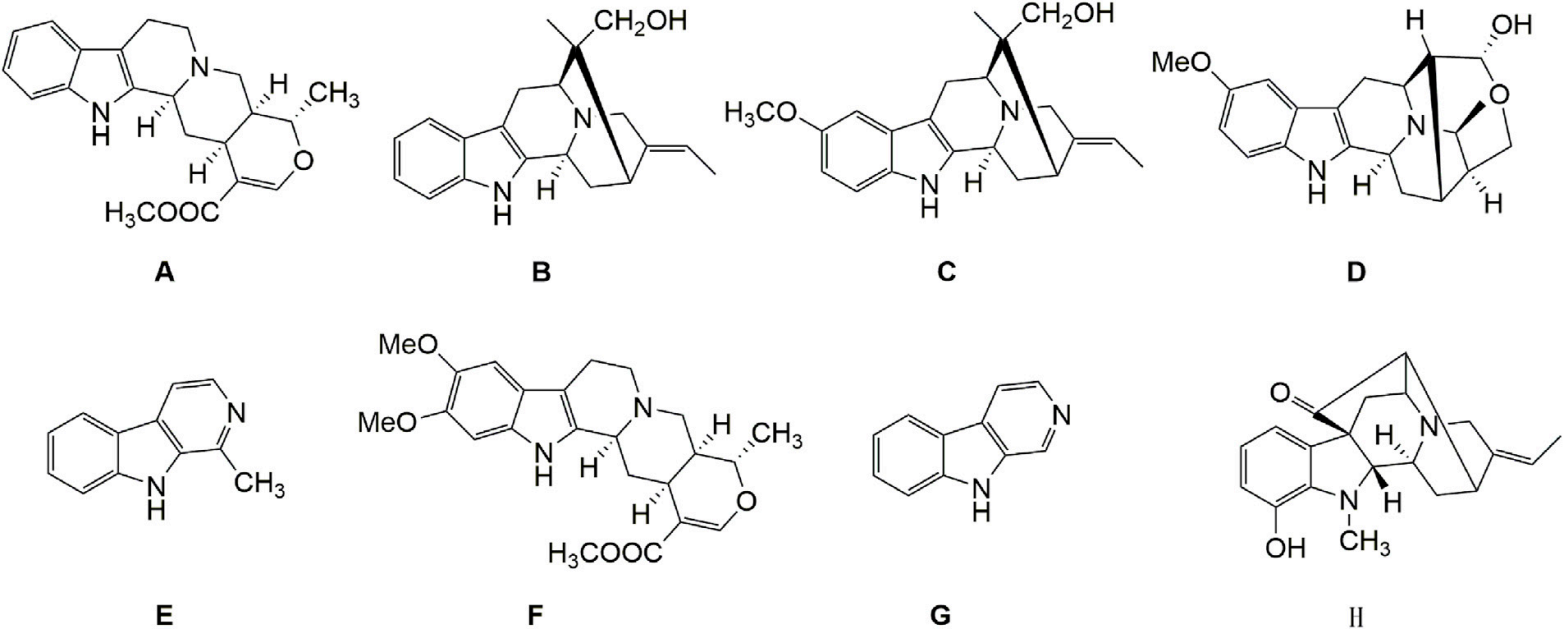
The structures of recognized compounds. **(A)** tetrahydropalmatine; **(B)** vellosimdinol; **(C)** rocodine; **(D)** vinnajorine D; **(E)** 1-methyl-β-Carboline; **(F)** isoreserpine; **(G)** β-Carboline; **(H)** raufomadine.

In our previous work, a large experimental database of the ^1^H chemical shift data of natural products was established and more than 200 indole alkaloids obtained from Apocynaceae were included (You et al., 2021). The MestReNova software was used to search ^1^H spectra of fraction 7, and the results were shown in Table 1. In order to obtain more accurate structural information, the search parameters are set as follows: the search display mode is spectrum search; the search method is Tree Similarity; and the other parameters are the default values, with 1000 points as the maximum score. As shown in Table 1, sabazin type compounds, including vellosimdinol, rocodine and vinnajorine D, had greater scores (765, 765 and 759), and the scores of 1-methyl-*β*-Carboline and *β*-Carboline were 754 and 735. Besides, two armaniline type compounds tetrahydropalmatine (784) and isoreserpine (736) and one armaniline type compound raufomadine (656) were also identified in the database. The structures of these compounds were shown in Figure 6. Combined the results with the biochemometric analysis, vellosimdinol, rocodine, vinnajorine D, 1-methyl-*β*-Carboline and *β*-Carboline are considered as potential chemosensitizing components.

**TABLE 1:** Searching ^1^H spectrum of Fraction seven from the database, and the following are the options with the highest scores and their corresponding compounds.

No.	Compounds	Scores
A	tetrahydropalmatine	784
B	vellosimdinol	765
C	rocodine	765
D	vinnajorine D	759
E	1-methyl-β-Carboline	754
F	isoreserpine	736
G	β- Carboline	735
H	raufomadine	656

### 2.4 Isolation and structural elucidation of the active compounds from RVA extract

To confirm the findings obtained by the chemometric analysis, the components in fraction seven and other fractions were isolated using silica gel column chromatography and HPLC. Their structures were elucidated by UV, NMR and MS as reported below. Five compounds (**3**–**7**) were obtained from fraction seven by column chromatography. Additionally, 16 compounds, including two new compounds (**1**, **2**), were isolated from other fractions. The structural elucidation of the new compounds is reported below.

**FIGURE 7:**
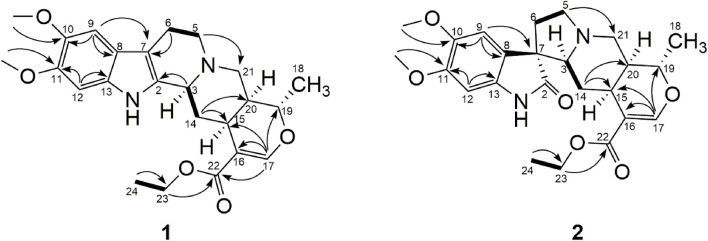
Key ^1^H–^1^H COSY (▬) and HMBC (H→C) correlations of compounds 1 and 2.

Compound **1**, isolated as yellow amorphous powder displayed the molecular formula C_24_H_31_N_2_O_5_ from HRESIMS [*m/z* 427.2224 ([M + H] ^+^) (calcd. For C_24_H_31_N_2_O_5_, 427.2227)]. Its UV absorption (254, 208 nm) was similar to that of isoreserpiline (Gupta et al., 2012). The ^1^H signals of compound 1 revealed the presence of two methyl proton signals [δ_H_ 1.31 (3H, *t*, *J* = 7.2 Hz) and *δ*_H_ 1.40 (3H, *d*, *J* = 6.2 Hz)], five methylene proton signals [*δ*_H_ 2.65 (1H, *m*), *δ*_H_ 1.43, (1H, *m*), *δ*_H_ 2.90 (1H, *m*), *δ*_H_ 2.65 (1H, *m*), *δ*_H_ 3.02 (1H, *m*), *δ*_H_ 2.54 (1H, *td*, *J* = 11.5, 4.5 Hz), *δ*_H_ 3.17 (1H, *dd*, *J* = 12.5, 1.7 Hz), *δ*_H_ 2.75 (1H, *dd*, *J* = 12.5, 3.9 Hz), *δ*_H_ 4.20 (2H, *m*)], four methine proton signals [*δ*_H_ 1.72 (1H, *m*), *δ*_H_ 2.77 (1H, *m*), *δ*_H_ 3.32 (1H, *m*), *δ*_H_ 4.46 (1H, *m*)], two aromatic proton signals [*δ*_H_ 6.91 (1H, *s*), *δ*_H_ 6.92 (1H, *s*)], a double bond proton signal *δ*_H_ 7.57 (1H, *s*), and two methoxy proton signals [*δ*_H_ 3.81 (3H, *s*), *δ*_H_ 3.83 (3H, *s*)]. The ^13^C NMR signals of compound **1** revealed the presence of an indole moiety (*δ*_C_ 96.7, 102.0, 107.9, 121.5, 132.5, 134.4, 145.7, 147.6), a carbonyl carbon signal (*δ*_C_ 169.2), two double bond carbon signals (*δ*_C_ 111.2, 156.8), two methyl carbon signals (*δ*c 14.7, 18.9), five methylene carbon signals (*δ*c 22.5, 34.8, 55.0, 56.8, 61.0), four methine carbon signals (*δ*c 32.7, 39.9, 61.9, 73.6), and two methoxy carbon signals (*δ*c 57.1, 57.2). Moreover, ^1^H and ^13^C-NMR data (Supplementary Table S1) indicated that a monoterpenoid moiety was present, which was similar to the compound isoreserpiline. The main difference between these two compounds in NMR spectra was that there was one more methylene carbon (*δ*_C_ 62.6) in compound **1**. Combined with the significant HMBC correlations (Figure 7) between H-23 and C-22, 24, a C-23 methylene group was established. From the evidence accumulated above, the structure of compound **1** was established (Figure 7) and was named Rauvolf A.

Compound **2**, isolated as yellow amorphous powder displayed the molecular formula C_24_H_31_N_2_O_6_ from HRESIMS [*m/z* 443.2176 ([M + H] ^+^) (calcd. For C_24_H_31_N_2_O_5_, 443.2177)]. The ^1^H signals of compound 2 revealed the presence of two methyl proton signals [*δ*_H_ 1.20 (3H, *t*, *J* = 7.1 Hz), *δ*_H_ 1.44 (3H, *d*, *J* = 6.3 Hz)], five methylene proton signals [*δ*_H_ 1.53 (1H, *dt*, *J* = 13.2, 3.5 Hz), *δ*_H_ 0.80 (1H, *q*, *J* = 12.0 Hz), *δ*_H_ 2.30 (1H, *m*), *δ*_H_ 1.96 (1H, *m*), *δ*_H_ 3.29 (1H, *td*, *J* = 8.8, 2.7 Hz), *δ*_H_ 2.40 (1H, *m*), *δ*_H_ 3.39 (1H, *dd*, *J* = 12.0, 2.0 Hz), *δ*_H_ 2.41 (1H, *m*), *δ*_H_ 4.08 (2H, *q*, *J* = 7.1 Hz)], four methine proton signals [*δ*_H_ 1.63 (1H, *m*), *δ*_H_ 2.43 (1H, *m*), *δ*_H_ 2.47 (1H, *m*), *δ*_H_ 4.37 (1H, *m*)], two aromatic proton signals [*δ*_H_ 6.63 (1H, *s*), *δ*_H_ 6.98 (1H, *s*)], a double bond proton signal *δ*_H_ 7.46 (1H, *s*), and two methoxy proton signals [*δ*_H_ 3.79 (3H, *s*), *δ*_H_ 3.84 (3H, *s*)]. The ^13^C NMR signals of compound 2 revealed the presence of an indole moiety (*δ*_C_ 97.2, 111.3, 126.1, 136.3, 146.0, 151.0), an ester carbonyl carbon signal (*δ*_C_ 168.9), a carbonyl carbon signal (*δ*_C_ 183.4), two double bond carbon signals (*δ*_C_ 111.4, 156.3), two methyl carbon signals (δc 14.6, 18.8), five methylene carbon signals (*δ*_C_ 31.3, 35.0, 54.4, 54.9, 60.7), four methine carbon signals (*δ*c 31.9, 39.4, 72.6, 73.7), a tertiary carbon signal (δc 58.8), and two methoxy carbon signals (*δ*c 56.8, 57.6). The NMR data (Table S1) indicated that the structure of compound 2 is similar to that of compound carapanaubine. The main difference between these two compounds in NMR spectra was that one methylene carbon (*δ*_C_ 60.7) was observed in the compound **2** (Reina et al., 2011). Combined with the significant HMBC correlations between H-23 and C-22, 24, a C-23 methylene group was established. From the evidence accumulated above, the structure of compound **2** was established (Figure 7) and was named Rauvolf B.

The structures of the five compounds (**3**–**7**) from fraction 7, and 14 compounds (**8**–**21**) from other fractions were determined by comparing their MS, UV, and NMR data with those reported in the literature. The known compounds were *β*-carboline (**3**) (Bratchkova et al., 2012), 1-methyl-*β*-carboline **4**) (Bratchkova et al., 2012), sarpagine (**5**) (Rukachaisirikul et al., 2017), lochnerine (**6**) (Kam et al., 1999), vellosiminol (**7**) (Mbeunkui et al., 2012), tetrahydroalstonine (**8**) (Achenbach et al., 1997), isoreserpiline (**9**) (Gupta et al., 2012), 10-methoxytetrahydroalstonine (**10**) (Gupta et al., 2012), carapanaubine (**11**) (Khatoon et al., 2022), isocarapanaubine (**12**) (Ahmad et al., 2010), N_a_-methylrauflorine (**13**) (Carlos et al., 2016), venoterpine (**14**) (Rukachaisirikul et al., 2017), mitoridine (**15**) (Kumara et al., 2019), yohimbine (**16**) (Zhan et al., 2020b), ajmaline (**17**) (Lim et al., 2014), vinmajorine D (**18**) (Zhang et al., 2016), 17-*epi*-vinmajorine D (**19**) (Zhang et al., 2016), peraksine (**20**) (Gao et al., 2015), and 17-*epi*-peraksine (**21**) (Arthur et al., 1968).

### 2.5 The chemosensitization effects of the compounds from fraction 7

According to the chemometric strategy, it was found that the fraction seven was the most active according to the previous study using the method. To verify the effectiveness of the strategy, the chemosensitive effects of compounds (**3**–**7**) from fraction seven on 5-FU against the proliferation of two colon cancer cell lines HCT-8 and LoVo were initially evaluated by the MTT assay. The 50% inhibition concentrations (IC_50_) of 5-FU were presented in Table 2. In case of HCT-8 cell, the combination of 40 μM compounds (**3**, **4**) and 5-FU significantly reduced the viability than the group treated by 5-FU only. However, the compounds **5**, **6** and **seven** could not increase the cytotoxicity of the 5-FU, and show no effect on the chemosensitization. In the case of LoVo cell, when in combination with the 5-FU, all the five compounds could increase its cytotoxicy of 5-FU. Among the compounds, compound **6** exhibited the best sensitization effect on LoVo cell. Notably, compound **3** and **4** showed considerable chemosensitization effect on both HCT-8 and LoVo cells. These results indicated that when compounds isolated from fraction seven combined with 5-FU, the amount of 5-FU needed to kill the cancer cells reduced andits efficacy against CRC cells was improved.

**TABLE 2:** The antiproliferative activity of compound **3**-**7** **combined with 5-FU against colorectal cancer cells**.

Compounds	IC50 (μM) for 48 h	
	HCT-8	LoVo
5-FU	8.47	19.3
3 + 5-FU	3.94	10.40
4 + 5-FU	5.09	15.99
5 + 5-FU	9.13	12.15
6 + 5-FU	8.55	5.20
7 + 5-FU	8.33	17.09

## 3 Conclusion

Bioassay-guided fractionation has an important role in the natural product discovery process. This methodology is an iterative methodology that alternates between chemical fractionation and bioassays. However, the use of bioassay-guided fractionation for isolation has limitations. One limitation for studying natural product mixtures is the difficulty in tying identified compounds to bioactive effects. Therefore, there is a need to guide the activity separation, and provide some necessary structural information at the same time. The method we established can obtain the structure information of the active compounds, while combining with the database can quickly obtain the compounds present in the active fractionations. In this study, a HPLC separation and chemometrics tools used ^1^H NMR data integrated with statistical models to highlight potential bioactive compounds with chemosensitization effect in the RVA extract. Regarding our results, combination of the ^1^H NMR-based metabolomic profiling with chemometric statistical analysis provides an advantageous approach to study the bioactive natural products in natural complex mixtures at a very early stage. Application of the method in bioactive compounds discovery makes both the initial stage, as well as subsequent oriented-isolation process simple and fast. However, the identified compounds and the isolated compounds do not completely coincide, due to the lack of comprehensive database and signal overlap in ^1^H NMR spectra. In the subsequent study, several approaches have been developed for obtaining accurate results, such as constructing a more comprehensive database, and combining pure chemical shift mapping and 2D NMR spectra with chemometric methods. Further directions of this work will be to apply the workflow to other bioactive extracts, in the context of drug discovery screening research.

## 4 Experimental section

### 4.1 Chemicals and reagents

High performance liquid chromatography (HPLC) grade acetonitrile was purchased from Tedia (United States). 5-Fluorouracil (5-FU) was purchased from Beijing Innochem Science & Technology co., LTD. Dimethyl sulfoxide (DMSO-*d*_6_) and tetramethylsilane (TMS) were obtained from Tenglong Weibo Technology (Qingdao, China). RPMI-1640 medium was acquired from M&C GENE TECHNOLOGY LTD. (Beijing, China), and fetal bovine serum was acquired from Gibco (ThermoFisher, Inc., United States). Purified water was obtained from a Milli–Q water purification system (Germany) and all other reagents were analytical grade. The dried roots of *R. vomitoria* were collected in Nanning, Guangxi Province, People’s Republic of China, on February 2018. A sample specimen (No. RV-WN-2018002) was deposited at School of Pharmacy Sciences, Shandong University.

### 4.2 Extraction and isolation

The dried roots of RVA (6 kg) were powdered and extracted three times (3, 3 and 3 h) with 95% ethanol. The extract was diluted with water and acidified with 5% H_2_SO_4_ to pH 1.0. The acid suspension was then extracted three times with petroleum ether and ethyl acetate, respectively. The resultant aqueous layer was subsequently neutralized with 10% NaOH solution and extracted with CH_2_Cl_2_, yielding 6.2 g crude alkaloidal extract.

The crude extract was applied to a preparative liquid chromatography to yield several fractions. The fractionation was accomplished on a Eclipse Plus C18 column (9.4 mm × 250 mm, 5 μm; Agilent Technologies, Inc., China) using an Agilent 1260 (Agilent Corporation, Santa Clara, CA, United States). The detection wavelength was set at 254 nm and the flow rate was 1.65 ml/min. The injection volume was 50 µl and the solvents used were 0.1% formic acid (A) and methanol: acetonitrile (1:1) (B). The gradient was as follows: from 0 to 7 min 10% B, from 7 to 100 min, linear gradient to 95% B. 18 fractions were sampled every 5 min using a fraction collector and were evaporated to dryness. The fractions were subjected to 1H NMR and biological activities evaluation.

### 4.3 Cell viability assay

Human colorectal carcinoma HCT-8 and LoVo cells were kindly provided by Cheeloo College of Medicine, Shandong University, and were maintained in RPMI-1640 medium supplemented with 10% fetal bovine serum, 100 U/mL penicillin, and 100 g/ml streptomycin. The cells were incubated in a humidifier at 37°C and 5% CO_2_.

HCT-8 and LoVo cells were seeded at 2 × 10^3^ cells/well into 96-well microplates and incubated with RVA extract and 5-FU for 24 h. MTT at a concentration of 0.5 mg/ml was added to each well. After 3 h incubation, the absorbance of the formazan crystals dissolved in DMSO was measured at 570 nm using a microplate reader (SPECTROstar Omega, BMG Labtech, Germany). The cell viability, directly proportional to the absorbance, was expressed as a percentage of untreated cells (control cells plus dimethyl sulfoxide less than 0.2%), and presented as the mean ± SD from three independent experiments.

### 4.4 Cell migration, colony formation, and ROS analysis

Cell migration ability was evaluated using the wound–healing assay. HCT-8 cells (1 × 10^5^ cells/well) maintained in a serum–free medium, were firstly incubated in 6–well plates for 24 h. Wounds of the cells were then created using a sterile pipette tip. Finally, the cells were treated with 5-FU only or in combination with RVA extract, respectively, for 48 h. The wound was photographed every 24 h with a bright–field microscope (IX71, Olympus, Japan) and the width was measured. Subtracting the scratch width at 0 h from the scratch width at other time points yields the migration distance.

The colony formation assay is performed to determine the inhibitory effect of cytotoxic agents on the colony-forming ability of a single cell (above 50 cells or more). HCT-8 cells (5 × 10^2^ cells/well) were seeded in 6-well plates and incubated for 24 h. Then, the cells attached to the plates were treated with 5-FU at 1 µM and RVA extract at 10.0 μg/ml, respectively, for 48 h. A fresh medium was added to the cells, which were incubated for another 14 days for colony formation. Finally, the colonies were stained with 0.1% crystal violet solution for 20 min and washed three times with PBS 1X. The number of colonies and their diameters were determined using the ImageJ.

Colon HCT-8 cell lines were seeded at 3 × 10^5^ cells/well into 60 mm well plates and treated with RVA extract and 5-FU at different concentrations for 48 h. After the treatments, 2′,7′-dichlorodihydrofluorescein diacetate (H2DCF-DA, Sigma-Aldrich, Europe) (5 μg/ml) was used to stain the cells for intracellular ROS measurement. The cells were then washed with PBS 1X and the fluorescent intensity were measured using flow cytometry after H2DCF-DA staining. Normalized ROS levels of cells treated with RVA extract only or combined with 5-FU were statistically compared to those of untreated cells.

### 4.5 Chemosensitization effects evaluation

The chemosensitization effect of each fraction of the RVA extract on 5-FU was estimated according to the previous method with some modifications (Ruiz-Torres et al., 2021). HCT-8 and LoVo cells were seeded, separately, in the 96-cell plate (5000 cells per well) and allowed to attach overnight in a humidified incubator containing 5% CO_2_ at 37°C. The cells were then treated with 100 μg/ml of each fraction and one μΜ 5-FU for 48 h. Surviving cells were quantified using Cell Counting Kit-8 (CCK-8) assay and the chemosensitization effect of each fraction was confirmed by a significant reduction in the number of viable cells. All experiments were performed in two independent trials with five plates per trial.

### 4.6 NMR analyses

The NMR samples were prepared, and all the experiments were performed as follows. Briefly, each fraction (3.6 mg) was dissolved in 600 μl of DMSO-*d*_6_ mixed with tetramethylsilane (TMS, 0.05% v/v), vortexed for 15 min, and filtered to remove the solid particles. The filtrate (550 μl) was then transferred to a 5 mm NMR tube. The NMR experiments were performed at 600 MHz on a Bruker Avance III spectrometer. NMR data processing were performed in Bruker TopSpin 3.6.2. The structure of the compounds was determined by comparison to references and NMR spectra.

### 4.7 Biochemometric data analysis

The key to this methodology was to propose a complete workflow, which was composed of four major steps. The first step was to obtain different fractions of the extract using HPLC. Secondly, the chemical profile of each fraction was acquired using ^1^H NMR analysis and their biological activities were evaluated. Then, we evaluated the links between the relative abundances of peak area in each fraction (structure information of the compounds in fractions) and their bioactivity, using chemometrics tools. The last step was to assign these signals to the probable bioactive substances using 2D NMR spectra. For statistical analysis, the matrix was firstly scaled by MestReNova (version 14.2, Mestrelab Research). Multivariate analysis was then performed using OPLS-DA method. In the OPLS-DA analysis, the chemosensitization effect of each fraction was added as a dependent variable. To achieve this purpose, a discrete variable for each fraction was determined as active or inactive in a binary classification using the following criteria: fractions with IC_50_ values lower than 50 μg/ml were considered as active, while those fractions with IC_50_ values higher than 50 μg/ml were considered inactive. Biochemometric analyses were performed separately for each cell (HCT-8 and LoVo). Cut-off values for both correlation (*p* [corr]) and covariance (w*) were applied to select the biomarkers candidates from the S plots by applying. Tentative identification of the corresponding compounds was performed based on the database.

### 4.8 Validation of the statistical model: Isolation of alkaloids with chemosensitization effects from the extract of RVA

To validate the OPLS-DA results, the isolation and structural identification of the compounds in the fractions from section 2.2 were carried out. For this purpose, Fraction seven was firstly selected according to the distribution variables plot. The dried and powdered Fraction 7 (50 g) was firstly fractionated by column chromatography with Sephadex LH-20 eluted with a mixture of chloroform and methanol (v:v, 1:1). Four fractions (F7.1 to F7.4) were obtained and then analyzed with successive silica gel columns in a gradient mode of chloroform-methanol. Five compounds named as **3**-**7** were isolated and characterized by NMR. Then, the chemosensitization effect of each compound was measured by the method as previously described. In addition, 14 known monoterpenoid indole alkaloids (**8**–**21**), together with two new compounds **1** and **2**, were obtained from other fractions of RVA extract.

Rauvomtine A **1**): A light yellow amorphous powder; IR (KBr) *ν*_max_ 3349, 2931, 2848, 1694, 1626, 1488, 1297, 1207 cm^−1^; ^1^H (600 MHz) and ^13^C NMR (150 MHz) spectral data in CD_3_OD, see Table S1; HRESIMS: *m/z* 427.224 [M + H]+(calcd for C_24_H_31_N_2_O_5_, 427.2227).

Rauvomtine B **2**): A light yellow amorphous powder; IR (KBr) *ν*_max_ 3262, 2945, 2791, 1702, 1627, 1502, 1312, 1209, 1190 cm^−1^; ^1^H (600 MHz) and ^13^C NMR (150 MHz) spectral data in CD_3_OD, see Table S1; HRESIMS: *m/z* 443.2176 [M + H]+(calcd for C_24_H_31_N_2_O_6_, 443.2177).

## Data Availability

The original contributions presented in the study are included in the article/Supplementary Materials, further inquiries can be directed to the corresponding authors.
