# Supplementary material for: Identification of chemosensitizing agents of colorectal cancer in Rauvolfia vomitoria using an NMR-based chemometric approach
Source: Front Chem. 2023 Jan 6;10:1069591. doi: 10.3389/fchem.2022.1069591 (PMC9852911; doi:10.3389/fchem.2022.1069591)
Supplement: Supplementary file 2 [file DataSheet1.docx]

Supplementary Material

**CONTENTS**

**Scheme 1** Extraction and separation of compounds from *R. vomitoria*

**Figure S1** 1H NMR (600 MHz, CD_3_OD) spectrum of compound **1**

**Figure S2** ^13^C NMR (150 MHz, CD_3_OD) spectrum of compound **1**

**Figure S3** ^1^H-^1^H COSY (600 MHz, CD_3_OD) spectrum of compound **1**

**Figure S4** HSQC (600 MHz, CD_3_OD) spectrum of compound **1**

**Figure S5** HMBC (600 MHz, CD_3_OD) spectrum of compound **1**

**Figure S6** NOESY (600 MHz, CD_3_OD) spectrum of compound **1**

**Figure S7** HRESIMS spectrum of compound **1**

**Figure S8** IR spectrum of compound **1**

**Figure S9** 1H NMR (600 MHz, CD_3_OD) spectrum of compound **2**

**Figure S10** ^13^C NMR (150 MHz, CD_3_OD) spectrum of compound **2**

**Figure S11** ^1^H-^1^H COSY (600 MHz, CD_3_OD) spectrum of compound **2**

**Figure S12** HSQC (600 MHz, CD_3_OD) spectrum of compound **2**

**Figure S13** HMBC (600 MHz, CD_3_OD) spectrum of compound **2**

**Figure S14** NOESY (600 MHz, CD_3_OD) spectrum of compound **2**

**Figure S15** HRESIMS spectrum of compound **2**

**Figure S16** IR spectrum of compound **2**

**Figure S17** The scheme of the different stages of the analytical process

**Table S1** ^1^H (600 MHz) and ^13^C (150 MHz) NMR data for compounds **1** and **2**.

**Scheme 1** Extraction and separation of compounds from *R. vomitoria*

**

**

**Fig S1** 1H NMR (600 MHz, CD_3_OD) spectrum of compound **1**

**Fig S2** ^13^C NMR (150 MHz, CD_3_OD) spectrum of compound **1**

**Fig S3** ^1^H-^1^H COSY (600 MHz, CD_3_OD) spectrum of compound **1**

**Fig S4** HSQC (600 MHz, CD_3_OD) spectrum of compound **1**

**Fig S5** HMBC (600 MHz, CD_3_OD) spectrum of compound **1**

**Fig S6** NOESY (600 MHz, CD_3_OD) spectrum of compound **1**


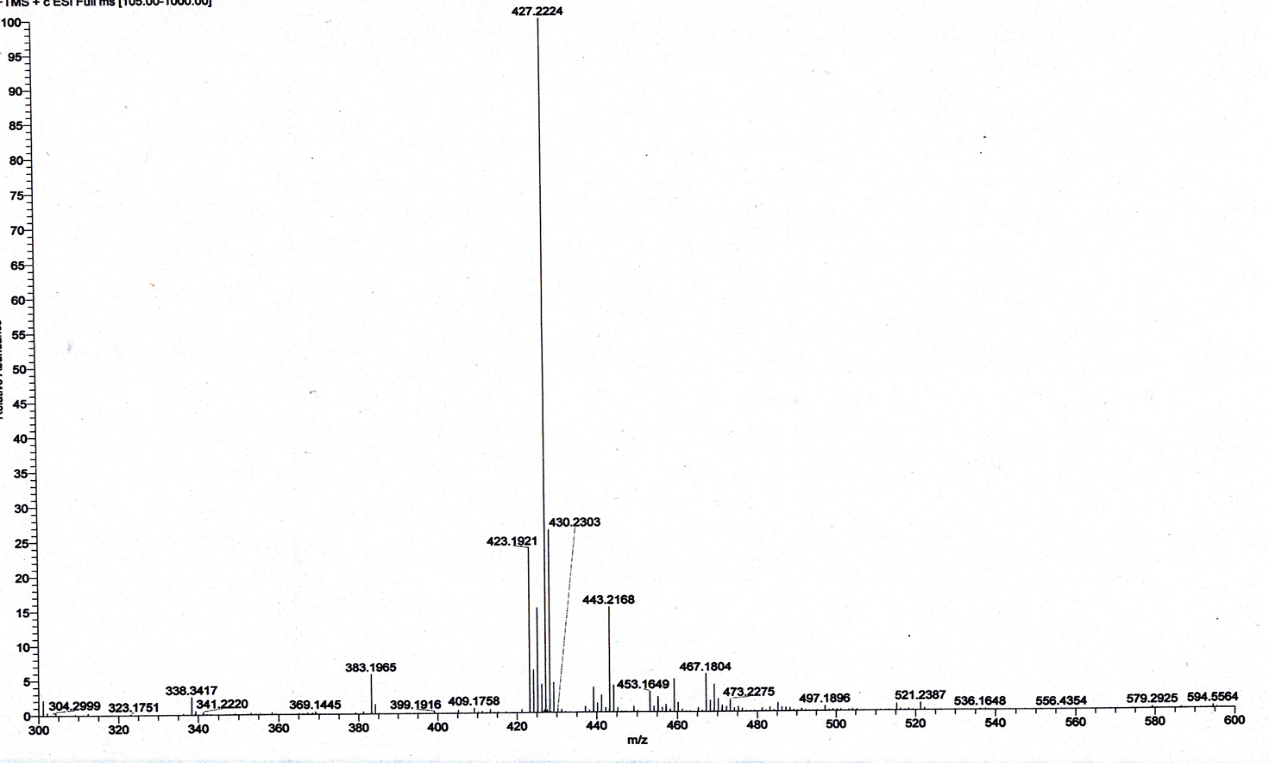


**Fig S7** HRESIMS spectrum of compound **1**


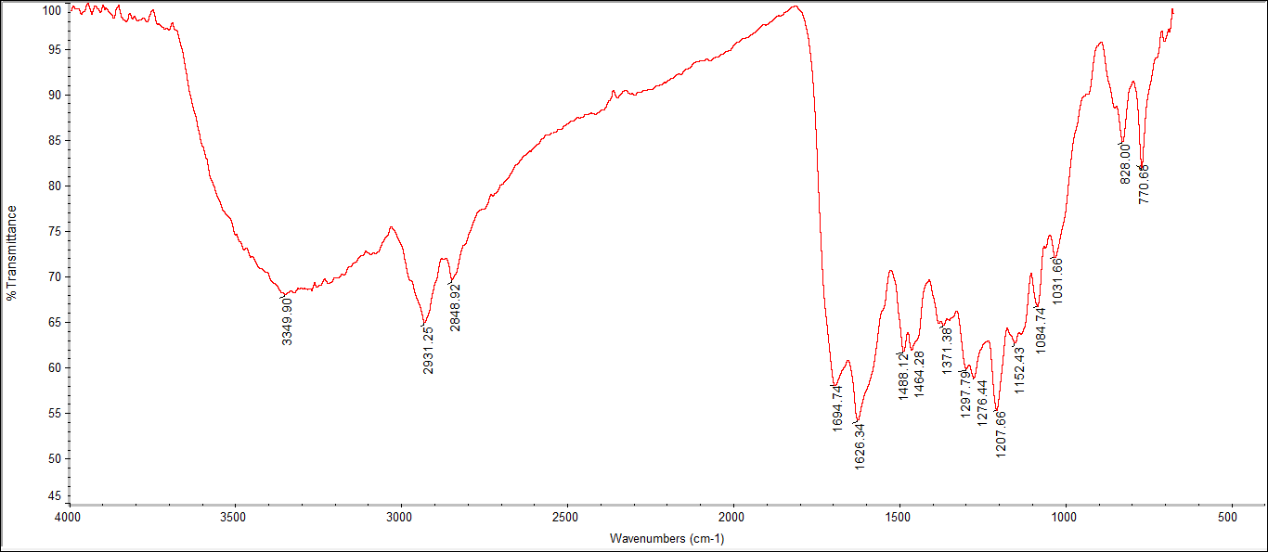


**Fig S8** IR spectrum of compound **1**

**Fig S9** 1H NMR (600 MHz, CD_3_OD) spectrum of compound **2**

**Fig S10** ^13^C NMR (150 MHz, CD_3_OD) spectrum of compound **2**

**Fig S11** ^1^H-^1^H COSY (600 MHz, CD_3_OD) spectrum of compound **2**

**Fig S12** HSQC (600 MHz, CD_3_OD) spectrum of compound **2**

**Fig S13** HMBC (600 MHz, CD_3_OD) spectrum of compound **2**

**Fig S14** NOESY (600 MHz, CD_3_OD) spectrum of compound **2**


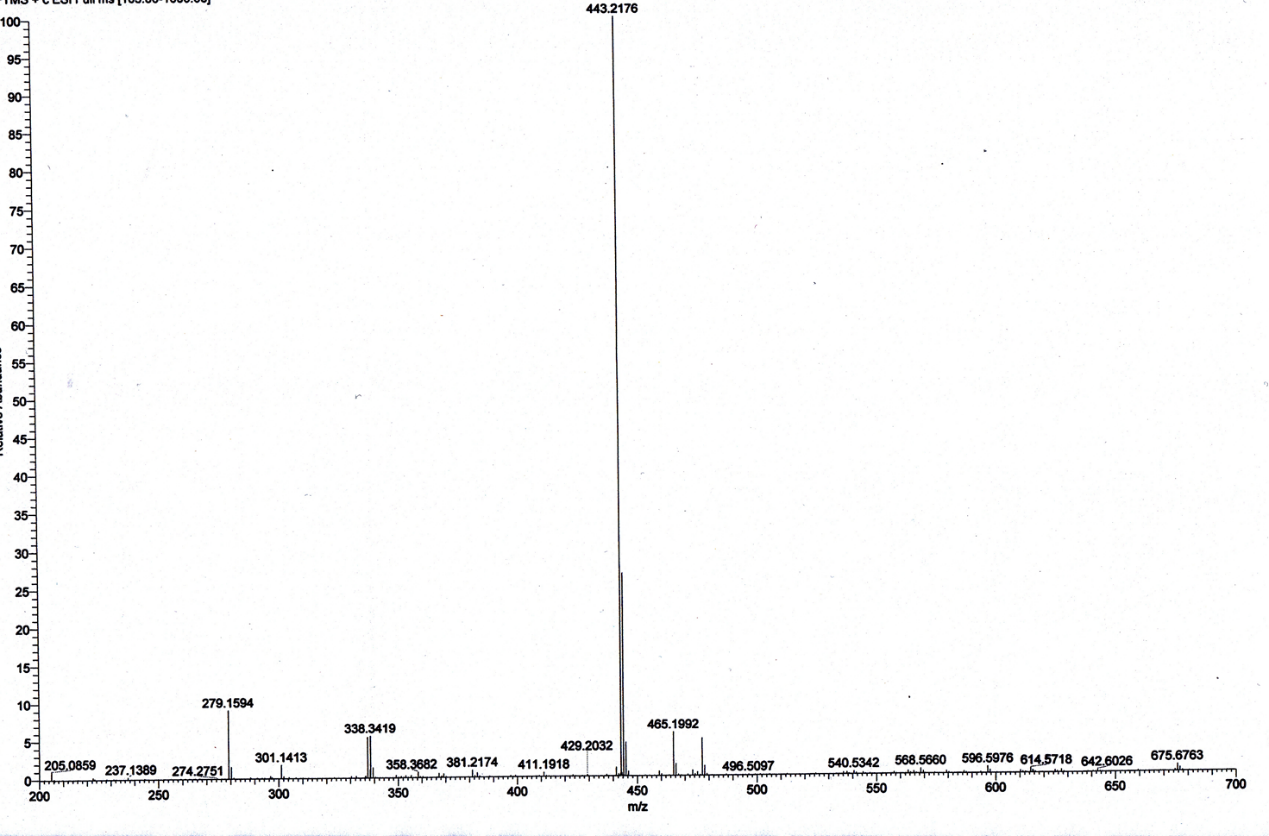


**Fig S15** HRESIMS spectrum of compound **2**

**
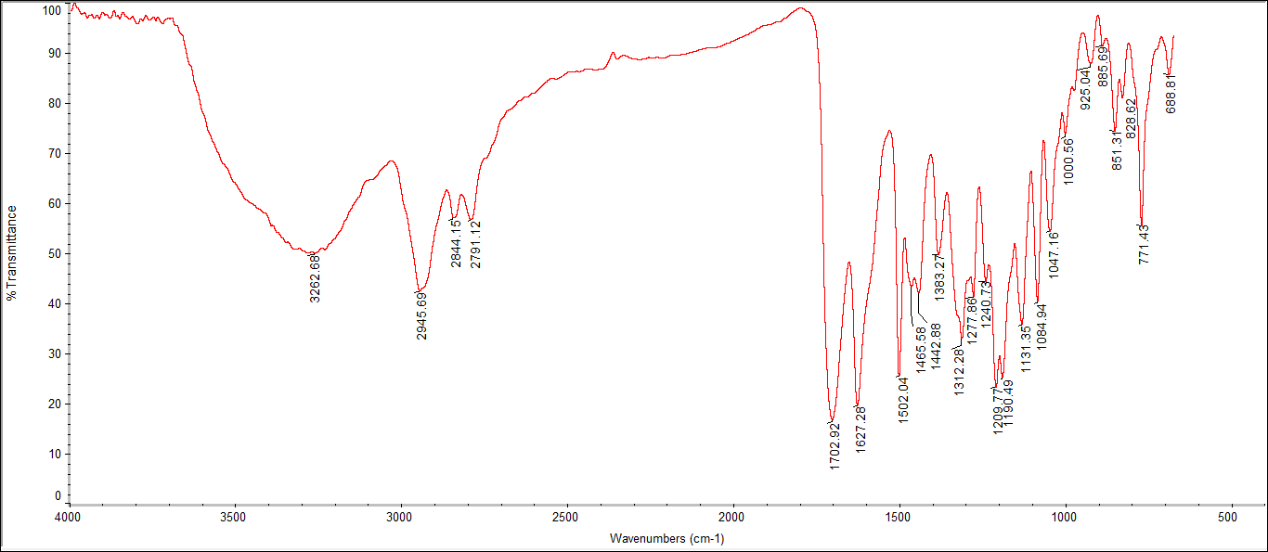
**

**Fig S16** IR spectrum of compound **2**

Figure S17. The scheme of the different stages of the analytical process.


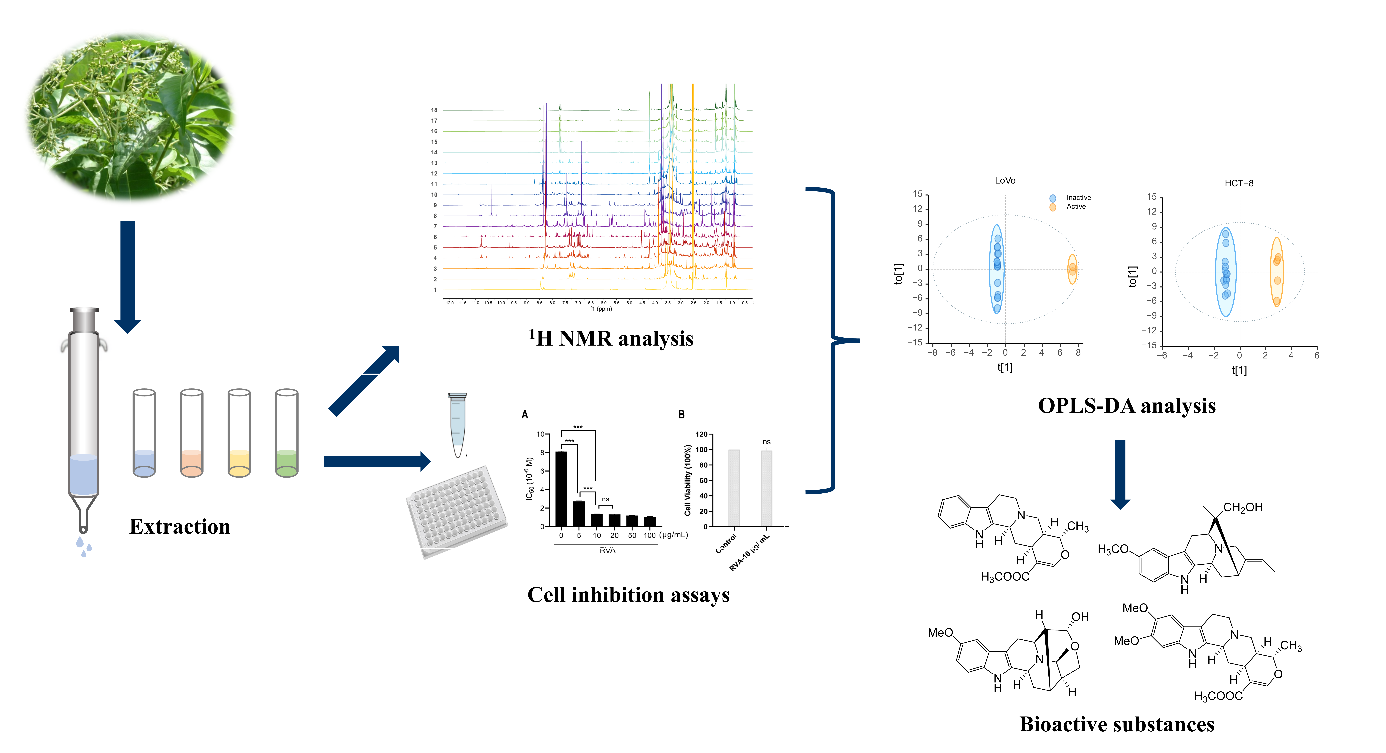


Figure S17 illustrates the scheme of the different stages of the analytical process. The key to this methodology was to propose a complete workflow, which was composed of four major steps. The first step was to obtain different fractions of the extract using HPLC. Secondly, the chemical profile of each fraction was acquired using ^1^H NMR analysis and their biological activities were evaluated. Then, we evaluated the links between the relative abundances of peak area in each fraction (structure information of the compounds in fractions) and their bioactivity, using chemometrics tools. The last step was to identify the probable bioactive substances to which these signals belong using 2D NMR spectra.

**Table S1**

^1^H (600 MHz) and ^13^C (150 MHz) NMR data for compounds **1** and **2**.

| No. | **1** | |  | **2** | |
| --- | --- | --- | --- | --- | --- |
|  | *δ*_C_ | *δ*_H_ |  | *δ*_C_ | *δ*_H_ |
| 1 |  |  |  |  |  |
| 2 | 134.4 |  |  | 183.4 |  |
| 3 | 61.9 | 3.32, *m* |  | 72.6 | 2.43, *m* |
| 4 |  |  |  |  |  |
| 5 | 55.0 | 3.02, *m* |  | 54.9 | 3.29, *td* (8.8, 2.7) |
|  |  | 2.54, *td* (11.5, 4.5) |  |  | 2.40, *m* |
| 6 | 22.5 | 2.90, *m* |  | 35.0 | 2.30, *m* |
|  |  | 2.65, *m* |  |  | 1.96, *m* |
| 7 | 107.9 |  |  | 58.8 |  |
| 8 | 121.5 |  |  | 126.1 |  |
| 9 | 102.0 | 6.92, *s* |  | 111.3 | 6.98, *s* |
| 10 | 147.6 |  |  | 146.0 |  |
| 11 | 145.7 |  |  | 151.0 |  |
| 12 | 96.7 | 6.91, *s* |  | 97.2 | 6.63, *s* |
| 13 | 132.5 |  |  | 136.3 |  |
| 14 | 34.8 | 2.65, *m* |  | 31.3 | 1.53, *dt* (13.2, 3.5) |
|  |  | 1.43, *m* |  |  | 0.80, *q* (12.0) |
| 15 | 32.7 | 2.77, *m* |  | 31.9 | 2.47, *m* |
| 16 | 111.2 |  |  | 111.4 |  |
| 17 | 156.8 | 7.57, *s* |  | 156.3 | 7.46, *s* |
| 18 | 18.9 | 1.40, *d* (6.2) |  | 18.8 | 1.44, *d* (6.3) |
| 19 | 73.6 | 4.46, *m* |  | 73.7 | 4.37, *m* |
| 20 | 39.9 | 1.72, *m* |  | 39.4 | 1.63, *m* |
| 21 | 56.8 | 3.17, *dd* (12.5, 1.7) |  | 54.4 | 3.39, *dd* (12.0, 2.0) |
|  |  | 2.75, *dd* (12.5, 3.9) |  |  | 2.41, *m* |
| 22 | 169.2 |  |  | 168.9 |  |
| 23 | 61.0 | 4.20, *m* |  | 60.7 | 4.08, *q* (7.1) |
| 24 | 14.7 | 1.31, *t* (7.2) |  | 14.6 | 1.20, *t* (7.1) |
| 10-OCH_3_ | 57.1 | 3.81, *s* |  | 57.6 | 3.79, *s* |
| 11-OCH_3_ | 57.2 | 3.83, *s* |  | 56.8 | 3.84, *s* |
